# Giant cell tumor of bone revisited

**DOI:** 10.1051/sicotj/2017041

**Published:** 2017-09-14

**Authors:** Andreas F. Mavrogenis, Vasileios G. Igoumenou, Panayiotis D. Megaloikonomos, Georgios N. Panagopoulos, Panayiotis J. Papagelopoulos, Panayotis N. Soucacos

**Affiliations:** 1 First Department of Orthopaedics, National and Kapodistrian University of Athens, School of Medicine, ATTIKON University Hospital 41 Ventouri Street 15562 Holargos Athens Greece

**Keywords:** Giant cell tumor of bone, Curettage, Cementation, Cauterization

## Abstract

Giant cell tumor (GCT) of bone is a locally aggressive benign neoplasm that is associated with a large biological spectrum ranging from latent benign to highly recurrent and occasionally metastatic malignant bone tumor. It accounts for 4–10% of all bone tumors and typically affects the meta-epiphyseal region of long bones of young adults. The most common site involved is the distal femur, followed by the distal radius, sacrum, and proximal humerus. Clinical symptoms are nonspecific and may include local pain, swelling, and limited range of motion of the adjacent joint. Radiographs and contrast-enhanced magnetic resonance imaging (MRI) are the imaging modalities of choice for diagnosis. Surgical treatment with curettage is the optimal treatment for local tumor control. A favorable clinical outcome is expected when the tumor is excised to tumor-free margins, however, for periarticular lesions this is usually accompanied with a suboptimal functional outcome. Local adjuvants have been used for improved curettage, in addition to systematic agents such as denosumab, bisphosphonates, or interferon alpha. This article aims to discuss the clinicopathological features, diagnosis, and treatments for GCT of bone.

## Introduction

Giant cell tumor (GCT) of bone is a relatively common, locally aggressive benign neoplasm that is associated with a large biological spectrum ranging from latent benign to highly recurrent and occasionally metastatic malignant potential [1]. It occurs most often in young adults, most commonly at the bones around the knee, followed by the distal radius, and the sacrum [1–4]. Different classifications have been proposed based on the histology, clinical and radiographic appearance, but they provide little prognostic information regarding the risk for local recurrence [3, 4]. Curettage alone has been the standard treatment for GCT, but it has been associated with a relatively high risk of local recurrence ranging up to 35–40% [1–4]. To reduce the risk for local recurrence, various local adjuvants such as cryosurgery, phenol, bone cement, zoledronic acid, hydrogen peroxide (H_2_O_2_) and argon beam, and systemic treatments such as bisphosphonates, interferon alpha (IFN-a), and denosumab have been reported, with variable results regarding the outcome, function, and complications for the patients [4, 5]. To enhance the literature, this article discusses the clinicopathological features, diagnosis, and treatments for the GCT of bone.

## Epidemiology

The GCT accounts for 4–10% of all primary bone tumors and approximately 20% of all benign bone tumors [6–8]. Patients with GCT present most often in their third decade of life, with approximately 80% of lesions occurring between 20 and 55 years of age [9]. A slight predilection for females has been reported, with a female-to-male ratio ranging from 1:1.1 to 1:1.5 [10]. Although GCT may affect all races, there is a strangely high prevalence (20–30%) for Chinese and southern Indian population, which, however, has not been explained to date [6, 10]. GCT typically occurs at the meta-epiphyseal region of long bones (75–90%), with approximately 84–99% of lesions extending to within 1 cm of subarticular bone. Most tumors occur at the bones around the knee (50–65% of all cases); the most common site is the distal femur (23–30%) followed by the proximal tibia (20–25%), distal radius (10–12%), sacrum (4–9%), and proximal humerus (4–8%) [10–13]. Atypical sites for GCT include the vertebral bodies and posterior elements of the mobile spine, the hands, feet, patella, and talus; atypical sites are common in multicentric GCT [1, 6, 10, 13, 14].

## Clinical presentation

Clinical symptoms are nonspecific and in order of decreasing frequency include pain, local swelling, and limited range of motion of the adjacent joint. Pain is usually present for several months and typically relieved by rest. Acute onset of pain may be associated with a pathologic fracture, which may occur at diagnosis in approximately 10–12% of patients [6, 14]. Neurological symptoms may be associated with spinal GCT [10].

The onset of symptoms in patients with GCT to the sacrum is generally insidious, with the patient typically complaining of slowly progressive symptoms evolving over a period of several months. The tumor might remain silent in its initial stages, being easily misdiagnosed or diagnosed with significant delay when it reaches a critical size. Symptoms, when present, usually comprise localized lower back pain, that may radiate to one or both legs, frequently mistaken for sciatica. Vague abdominal discomfort, early satiety, a progressive change in bowel or bladder habits, and sexual dysfunction have also been reported [15–17].

## Classification

**Figure 1.**
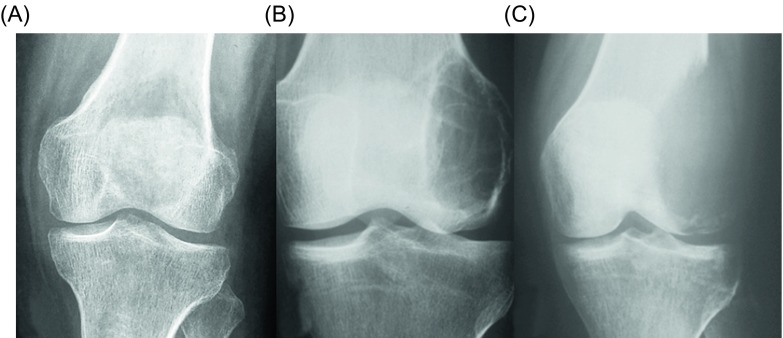
Campanacci et al. [3] grading system for GCT that is based on the radiographic appearance of the tumors. (A) Grade 1 (latent), (B) grade 2 (active), and (C) grade 3 (aggressive).

Numerous classification systems have been proposed over the years [18–22]. Jaffe et al. [18] classified GCT as benign, aggressive, and malignant based on the histological appearance of the stromal cells and the number of giant cells and mitoses. Nonetheless, the histological staging system of Jaffe and its prognostic value of this grading had been disputed. Campanacci et al. [3] classified the GCT into three grades depending on their radiographic appearance: a grade 1 lesion (latent) has a well-defined margin and an intact cortex; a grade 2 lesion (active) has a relatively well-defined margin but no radiopaque rim, and the cortex is thinned and moderately expanded; and a grade 3 lesion (aggressive) has indistinct borders and cortical destruction (Figure 1). Enneking et al. [19] proposed a clinico-radiological classification of three stages for benign bone tumors including GCT: stage 1 (latent) refers to a confined totally by bone, asymptomatic, inactive on bone scan, histologically benign lesion; stage 2 (active) refers to an expanded cortex with no breakthrough, symptomatic (often with a pathologic fracture), active on bone scan, histologically benign lesion; stage 3 (aggressive) refers to a rapidly growing mass, cortical perforation with soft tissue mass, may metastasize, symptomatic, extensive activity on bone scan, histologically benign; and stage IV (malignant) refers to a sarcomatous lesion contiguous with a benign GCT. The Campanacci grading system for GCT is similar to that proposed by Enneking for benign bone tumors overall.

It has been suggested that the GCT should be preferably classified as per Campanacci et al. [3] as this classification scheme may more easily guide treatment; grade 1 and grade 2 lesions should be treated with intralesional curettage, and grade 3 lesions with en block resection and reconstruction, if necessary [20]. However, it is doubtful whether these classifications accurately assess the aggressiveness of GCT, or provide reliable prognostic significance in terms of local recurrence rates and functional results. More importantly, they do not seem to provide valuable guidelines for decision-making on surgical treatment [21]. Definitely, no correlation exists between the grading systems and the incidence of local recurrence or metastases [3, 18–22].

## Imaging

Radiographs and contrast-enhanced magnetic resonance imaging (MRI) are the standard imaging modalities for the diagnosis of GCT. Computed tomography (CT) can be used to assess cortical thinning and pathologic fractures, and to monitor fracture consolidation. The typical radiographic features of GCT include a purely osteolytic lesion with a geographic type of bone destruction, [22] a well-defined but nonsclerotic margin, eccentric location, extension to the subchondral bone, closed physes [23]. Nonaggressive tumors exhibit a prominent trabeculation with no cortical expansion or soft tissue mass, whereas aggressive tumors exhibit a lack of trabeculation, with expansion or destruction of the cortex and an associated soft tissue mass [22, 23].

On MRI, GCT usually shows a low to intermediate signal intensity on T1 and a high signal on T2-weighted images. The intramedullary portion of the tumor is best seen on T1, whereas its extraosseous component is more clearly observed on T2-weighted images. After intravenous injection of gadolinium, heterogeneous enhancement of the tumor is observed. It has to be mentioned though that some reports indicate that certain cases of GCT containing large amounts of hemosiderin may show different MRI characteristics [24]. The MRI is also effective in demonstrating subchondral breakthrough and tumor extension to the adjacent joint [25]. Fluid levels in the tumor have been reported in 10–14% of patients and are considered to be secondary to an aneurysmal bone cyst (ABC) component [14, 26–28]. Dynamic contrast-enhanced MRI with intravenous gadolinium administration shows early and rapidly progressive enhancement followed by contrast washout [16].

Radionuclide bone scan rarely provides additional information, because the degree of tracer uptake does not correlate with the histologic grade of the tumor [27]. However, bone scan may help to detect multiple foci, if multicentric disease is clinically suspected. In a study by Hudson et al. [27], an abnormal uptake pattern was found in 49% of cases, resembling a doughnut that is intense uptake around the periphery with relatively little activity in the central portion of the tumor. The authors contended that this appearance was due to uptake of the bone-seeking radiopharmaceutical agents predominantly by reactive new bone or by hyperemic bone around the tumor, with the tumor tissue itself retaining little tracer. Occasionally, an increased tracer activity can be detected across the adjacent joint. This phenomenon may be due to increased blood flow and to increased bone turnover secondary to disuse osteoporosis [29, 30].

Five to ten percent of GCT may undergo malignant transformation [31, 32]. However, malignant GCTs are not accompanied by additional or specific imaging characteristics, therefore, they cannot be diagnosed radiographically. On the other hand, the radiographic presentation of GCT complicating Paget’s disease is usually that of an expansile lytic lesion, frequently accompanied by a soft tissue mass [33, 34].

## Diagnosis

Biopsy tissue sampling for histological examination, diagnosis, classification, and grading is necessary for GCT as for any bone and soft tissue tumor. The goal of biopsy is to obtain a diagnostic tissue sample without complications, tumor spread, and compromise of future treatments. As a rule, all lesions should be biopsied as if they were malignant [35, 36]. Traditionally, open biopsy has been the biopsy technique of choice for musculoskeletal tumors, providing adequate material for histological and immunohistochemical studies, resulting in a higher rate of accuracy compared with closed biopsy. Currently, imaging-guided closed biopsy with ultrasonography or CT is the gold standard for musculoskeletal tumors because of low cost, low risk of tumor spread and contamination, and minimal invasiveness for the patient. Imaging-guided closed biopsy increases the accuracy and reduces the risk of complications of the biopsy, especially for deep-seated tumors. An open biopsy is indicated when (1) a repeat closed biopsy is not diagnostic or is inconclusive, (2) an adequate tissue sample cannot be obtained with closed biopsy, and (3) the result of closed biopsy does not correlate with the clinical presentation and imaging findings [35, 36].

Chondroblastoma is an epiphyseal lesion that is classically included in the differential diagnosis of GCT. Their epiphyseal location and histologic characteristics are similar. However, GCT is almost exclusively seen in skeletally mature patients, while chondroblastoma tends to occur in skeletally immature patients. Furthermore, the epicenter of GCT lies within the metaphysis. Although an epiphyseal or apophyseal location is classic for chondroblastoma and extension into the metaphysis may be seen, purely metaphyseal or diaphyseal chondroblastomas have been reported. On imaging, GCT and chondroblastoma have similar features, including extensive perilesional edema on MRI. Therefore, histology is required to differentiate these lesions. Chondroblastoma shows typical round or polygonal mononuclear cells, chondroid matrix, and calcifications; in contrast, GCT has elongated cells that are clustered together, while calcifications and chondroid matrix are absent. Treatment of both lesions remains the same [1, 6, 10, 13, 14].

## Pathology

**Figure 2.**
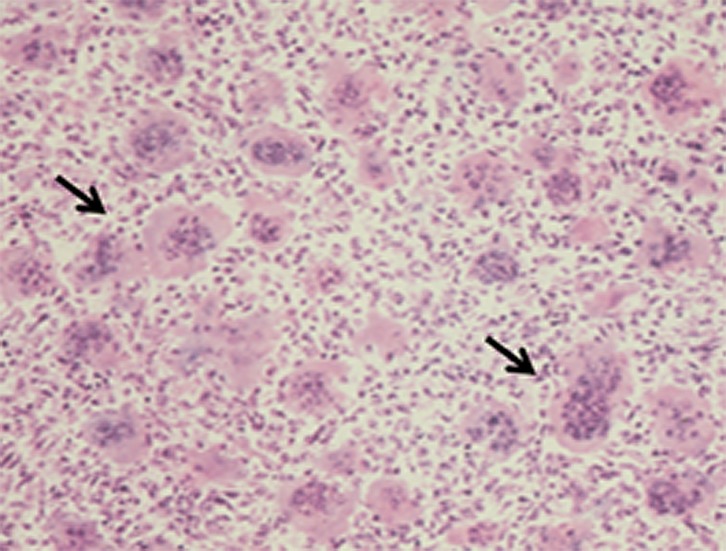
High power histopathologic image shows the characteristic multinucleated giant cells of the GCT (arrows).

Macroscopically, GCT usually represents soft, friable, fleshy, red-brown masses with yellowish areas. The cortex may or may not be involved initially, but it can be ultimately involved, with the original bone contour expanded or destroyed. There may be evidence of hemorrhage, hemosiderin deposition, cyst formation, necrosis, and pathologic fracture [1]. A secondary ABC may be present in 10–14% of GCT of bone cases [16]. Microscopically, the basic pattern of GCT is that of a moderately vascularized stroma with oval or plump, spindle-shaped mononuclear cells uniformly interspersed with multinucleated giant cells (Figure 2) [1]. The spindle-shaped mononuclear cells have poorly defined cytoplasm, spindle-shaped nuclei and show variable degrees of mitotic activity. They are thought to represent the proper neoplastic cell population [16]. The multinucleated, osteoclast-like giant cells have eosinophilic cytoplasm, vesicular nuclei and are thought to constitute a reactive cell population in the context of the tumor [16].

From a molecular biology point, receptor activator of nuclear factor kappa B (RANK) ligand (RANKL) is highly expressed by the neoplastic mononuclear stromal cells. It has been shown that the RANK-RANKL interaction and the macrophage colony-stimulating factor (M-CSF) play an important role in osteoclastogenesis by stimulating recruitment of osteoclastic cells from blood-borne mononuclear osteoclast precursor cells that differentiate into multinucleated osteoclast-like giant cells [37–39]. Cytogenetically, the most common chromosomal aberrations in GCT (50–70%) are telomeric associations and chromosomal end-to-end fusion [40, 41]. Telomere length maintenance is thought to be an important key factor in the pathogenesis of GCT [42]. Recently, a driver mutation has also been identified in H3F3A, in 92% of GCT of bone cases [43]. Furthermore, allelic losses of 1p, 9q, and 19q are common in primary, recurrent, and metastatic GCT [40]. Mutations of TP53 and HRAS are seen in secondary malignant GCT, probably playing a role in malignant degeneration [44, 45].

## Biologic behaviour

Approximately 25% of GCT are considered to be locally aggressive on clinical and imaging grounds [46]. These tumors show extensive bone destruction, cortical expansion, and soft tissue invasion [47]. One of the major issues with GCT is the propensity for local recurrence. After curettage alone, the local recurrence rates range from 25% to 35%, typically within two or three years [4, 47]. Neither local aggressiveness nor recurrence has been associated with any specific histologic findings [1]. GCT has a 2–5% incidence of metastasizing to the lungs, with the risk being greater in case of recurrent tumors, at an average of 3–4 years after initial diagnosis and index treatment [9, 48]. Pulmonary metastases in GCT, sometimes called benign pulmonary implants, are typically slow growing and usually amenable to surgical resection with a prospect for cure [49, 50]. Even though some patients might succumb as a result of multiple lung lesions, prognosis is favorable in more than 70% of patients, and some metastatic foci may resolve spontaneously [2, 51–61].

Currently, there are no reliable predictors of local recurrence or metastatic disease [10, 52–58]. The prognostic significance of a pathologic fracture in patients with GCT is controversial. It has been suggested that a pathologic fracture is associated with a poorer outcome in patients with a GCT of the bone, in terms of functional outcomes, recurrence rates, complications, and survival. However, a recent meta-analysis found no difference in local recurrence rates between patients who have a GCT of bone with and without a pathologic fracture at the time of presentation [62]. Therefore, the presence of a pathologic fracture should not preclude the decision to perform curettage as carefully selected patients who undergo curettage can have similar outcomes in terms of local recurrence to those without such a fracture [62].

A recent array comparative genomic hybridization study of 20 frozen tumors showed that 20q11.1 is frequently amplified in GCT, and its presence correlates with the occurrence of metastatic disease [52]. True malignant variants of GCT have also been reported. Kransdorf and Murphey [10] described a modification of a classification for malignant GCT previously reported by Mirra et al. [53]. They distinguished benign metastasizing GCT, which corresponds to the previously described disease with occasional lung nodules, true malignant GCT, which are defined as high-grade sarcomas arising in GCT (primary) or at the site of a previously documented GCT (secondary), and giant cell-rich sarcomas, which most commonly occur in association with other entities such as severe polyostotic Paget’s disease. Secondary malignant GCTs are the most common malignant variants, accounting for approximately 87% of such cases [10]. A history of previous radiation therapy is reported in 76% of patients with secondary malignant GCT, usually after a delay of 10 or more years [54]. With the decline in use of radiation therapy for GCT, the incidence of radiation-induced sarcomas has decreased significantly.

The presence of more than one primary GCT in the same patient is rare [55]. Less than 1% of GCT are multicentric or multifocal lesions [56], which may present synchronously (developing simultaneously or within a period of six months), or metachronously (second tumor appearing six months after diagnosis of the first) [57]. Multicentric involvement tends to be more clinically aggressive, and, unlike the solitary lesions, multicentric GCT has a propensity for atypical sites such as the vertebral bodies and posterior elements of the mobile spine, and the small bones of the hands and feet, patella and talus [1, 6, 10, 13, 14, 55–58]. Patients with multicentric lesions tend to be younger than those with lesions elsewhere [58].

## Surgical treatment

**Figure 3.**
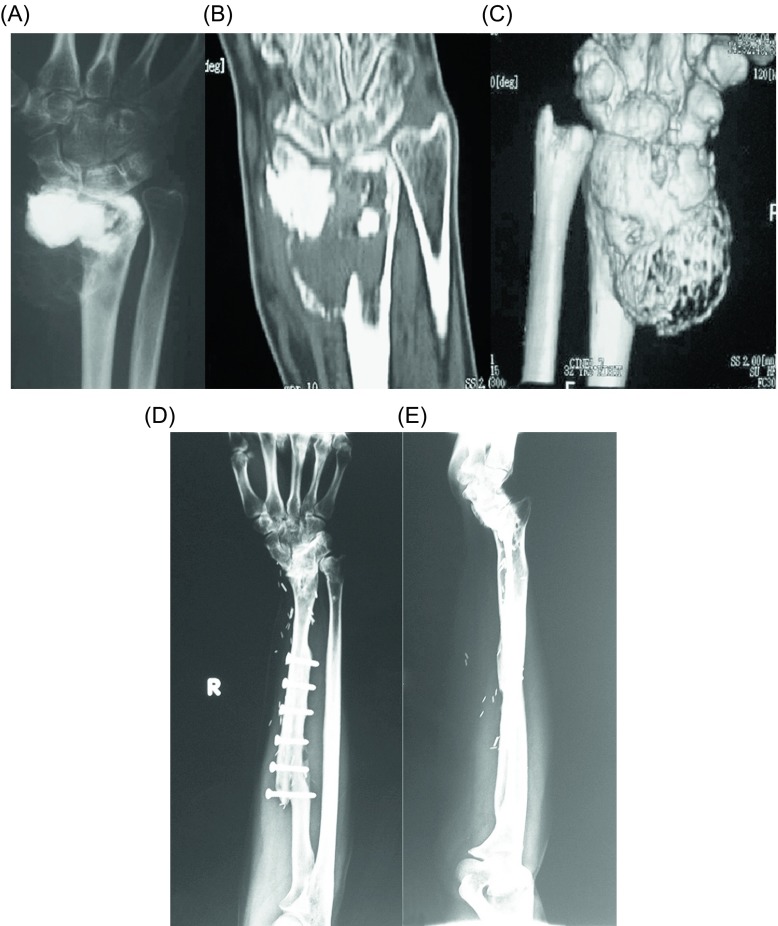
(A) Anteroposterior radiograph, (B) coronal CT, and (C) three-dimensional CT reconstruction of the right wrist of a 40-year-old man with a recurrent GCT of the distal radius after curettage and PMMA cementation. Wide resection and free vascularized fibula graft distal radius reconstruction were done. (D) Anteroposterior and (E) lateral radiographs of the right wrist show no evidence of local tumor recurrence at 16-year follow-up.

**Figure 4.**
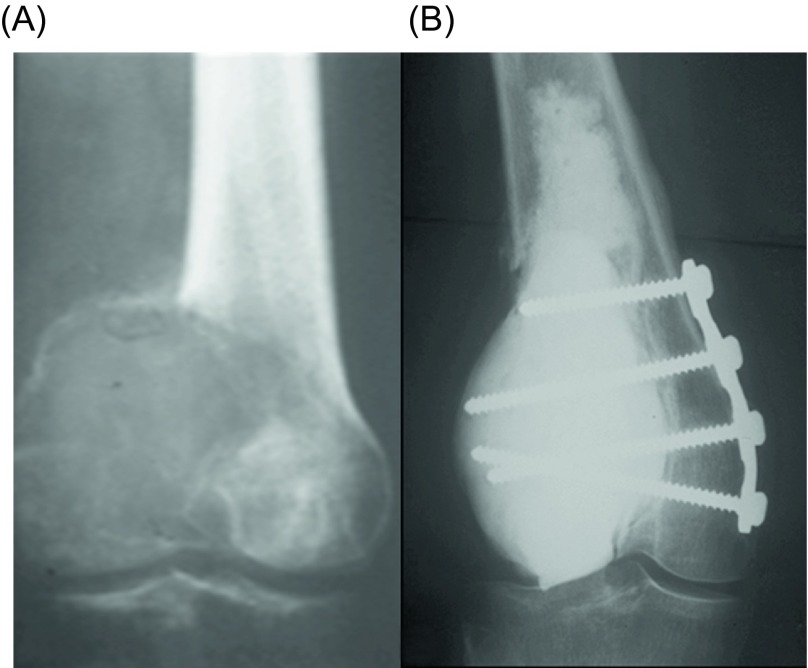
(A) Anteroposterior radiograph of the left knee of a 46-year-old man with a GCT of the medial femoral condyle. (B) Anteroposterior radiograph of the left knee seven years after curettage, cauterization, and cementation, in addition to short plate osteosynthesis shows no evidence of local tumor recurrence.

Surgical treatment is the treatment of choice for GCT. Depending on the involvement of the articular surfaces, the tumor can be removed either by resection (Figure 3) or with curettage (Figure 4), with or without local adjuvants. Surgical outcomes are optimal when the tumor is removed to tumor-free margins, with minimal surgical morbidity and an acceptable functional outcome. Resection with wide (microscopically negative) margins has been associated with few or no recurrences ranging from 0% to 16%, but a poor functional outcome and greater surgical morbidity [16]. Compared to en bloc resection, curettage presents higher recurrence rates (12–65%), but less morbidity and functional impairment for the patients [6, 46, 59, 60]. Therefore, it has been the mainstay of treatment for the majority of patients with Enneking stage I or II lesions. Recurrence after curettage is mostly diagnosed within two years of the index procedure [61]. Wide excision is usually reserved for more aggressive tumors with extraosseous extension, unresectable or multiply recurrent tumors.

**Figure 5.**
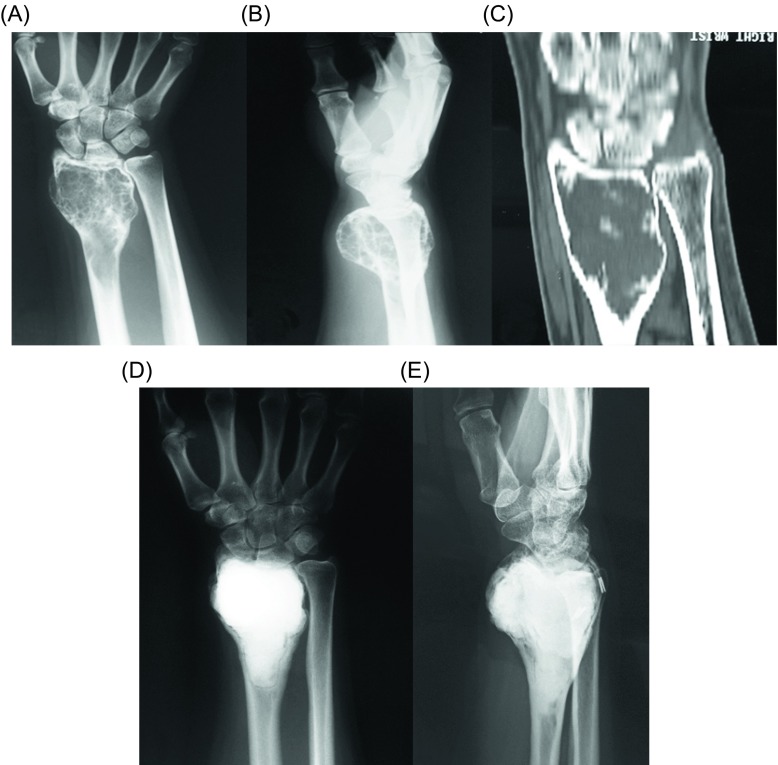
(A) Anteroposterior and (B) lateral radiographs, and (C) coronal CT of the right wrist of a 43-year-old man with a GCT of the distal radius. Curettage, cauterization, and PMMA cementation were done. (D) Anteroposterior and (E) lateral radiographs of the right wrist show no evidence of local tumor recurrence at eight-year follow-up.

Curettage can be performed alone or combined with local adjuvants (Figure 5). Curettage alone has the worst recurrence rates (mean: 42%; range: 21–65%) [46, 63–68]. Local adjuvants including cementation with polymethyl methacrylate (PMMA), alcohol, phenol, hydrogen peroxide, zinc chloride, cryoablation with liquid nitrogen, speed burr drilling, local application of zoledronic acid, and combinations have reduced local recurrence rates [46, 63–91]. Curettage with PMMA has been associated with local recurrence rates of 0–29% [46, 61, 63–65, 68–75, 90, 91]; when combined with local phenol application the local recurrence rates are 3–33% [46, 61, 63–65, 69, 71–74]. Local recurrences can be treated with repeat curettage, phenol, and PMMA, with re-recurrence rates of 9–34% (mean: 19%) [61, 64–67, 72, 73, 76–80]. Cryoablation with liquid nitrogen is associated with local recurrence rates of 8–42% (mean: 21%) and 0–20% (mean: 6%) when combined with bone grafts and PMMA [60, 65, 70, 72, 81–99].

During curettage, an osseous window is osteotomized in the cortex, the size of which depends on the tumor size; in general, it should be of adequate size for optimal curettage. Through this window, the surgeon should have full visibility of the tumor cavity, in order to curette the tumor entirely, without risking an iatrogenic fracture. Curettes of different sizes are used to remove as much of the lesion as possible and supplemented by high-speed burring of cavity. Phenol-induced osteonecrosis is limited to a depth of 1.5 mm, thereby reducing the risk of fracture, but has a rate of recurrence of approximately 20–30% [91, 92]. Liquid nitrogen produces osteonecrosis of the tumoral bed, which is 1–2 mm deep; three cycles of rapid freezing (−50 °C) and slow thawing (20 °C) are usually needed to increase margins up to 2 cm that is comparable with marginal resection [83, 84]. Filling the cavity with PMMA, hypothetically lowers recurrence risk, due to cement’s hyperthermic properties. Heat created during cement polymerization can sterilize the tumor wall (3–5 mm deep) and augment stability [91]. However, the role of PMMA for tumor necrosis has not been validated; certainly, PMMA provides immediate mechanical support, early mobilization and facilitates early detection of local recurrences [46, 63].

The use of local adjuvants is not without complications. Chemical burns can occur by phenol, if the application is not carefully performed, and special attention must be given to the neighboring neurovascular structures and soft tissues [78, 89]. Postoperative fracture, skin necrosis, transient nerve palsy, and infection are some of the complications reported with liquid nitrogen ablation (rates: 12–50%) [81, 85, 93]. Adequate monitoring of freezing temperatures and prophylactic fixation in selected cases have decreased fracture rates significantly from 25–50% to 0–7% [16]. In spite of the positive results and low recurrence rates, the high fracture risk due to difficult control of the depth of the induced osteonecrosis prevents this procedure from becoming the method of choice [92]; PMMA cementation still remains the preference for filling the defect of curettage and as local adjuvant. Complications of PMMA cementation range from 13% to 25% and include cement leakage into joints or surrounding soft tissues and osteoarthritic changes [46, 61–64].

## Systemic agents

New pharmaceutical treatments have been introduced for lesions or for patients in whom surgical treatment is not feasible. If GCT is initially inoperable, neoadjuvant systemic targeted therapy may facilitate intralesional surgery at a later stage, avoiding a more invasive surgery. Current understanding of the molecular biology of GCT and understanding of the involvement of the RANK/RANKL pathway in its pathogenesis have recently led to the increased use of denosumab [16, 94].

Denosumab is a human monoclonal antibody (immunoglobulin G2, IgG2) that targets and binds RANKL with high affinity and specificity, preventing the activation of its receptor, RANK, on the surface of giant cells, osteoclast precursors, and osteoclasts. Prevention of the RANK/RANKL interaction inhibits osteoclast formation, function, and survival, thereby decreasing bone resorption in GCT [94]. Denosumab has recently been approved by the United States (US) Food and Drug Administration (FDA) (June 2013) and by the European Medicine Agency (EMA) (September 2014) for the treatment of adults and skeletally mature adolescents with GCT that is unresectable or where surgical resection is likely to result in severe morbidity [94]. Recent studies have shown that GCT responds well to treatment with denosumab. An open-label phase 2 study showed that out of 100 patients with a planned surgery at baseline only 26 were operated, after they had been pretreated with denosumab; 74 patients had no surgery at all, and only three patients underwent a major surgery out of the 44 who were planned to be treated at baseline with this method [95]. The 2014 ESMO (European Society for Medical Oncology) guidelines mention that denosumab may be used to achieve cytoreduction, allowing potentially curative surgery, or also in unresectable and metastatic disease, where treatment needs to be maintained to avoid progression [5]. Long-term treatment may be required for long-term local control of GCT. The most important side effects of denosumab are headache and bone pain (1–10%), osteonecrosis of the jaw (1–2%), hypocalcemia and hypophosphatemia (< 0.01%) [16].

Bisphosphonates bind to bone mineral matrix, and are thought to inhibit GCT-derived osteoclast formation, migration, and osteolytic activity at sites of bone resorption, as well as to promote apoptosis of osteoclasts [16, 94]. In most reported inoperable tumors, stabilization of local and metastatic disease was achieved [16, 94]. It has been shown that nitrogen-containing bisphosphonates induce apoptosis in both giant cells and stromal cells *in vitro* [96]. In a case-control study, pamidronate and zoledronate significantly reduced local tumor recurrence (4.2% vs. 30% in the control group, *p* = 0.056) and controlled disease progression when used orally or intravenously as adjuvant therapy to intralesional curettage [97]. In 25 patients with recurrent and metastatic GCT treated with bisphosphonates, control of the disease was achieved in most cases refractory to conventional treatment [98]. However, further evidence is needed for definitive important conclusions to be drawn.

The increased expression of several angiogenic growth factors observed in GCT led to the use of IFN-a as an anti-angiogenic agent to control local and distant disease, however, with mixed results [94, 99]. The first use of IFN-a was in 1995. Pegylated (PEG)-IFN has also been shown to have anti-GCT activity [94]. Currently, many questions remain regarding the IFN therapy for GCT. Standardized treatment regimens need to be established and studied through multi-institutional clinical protocols to determine the effectiveness of IFN therapy [99].

## Conclusions

GCTs are locally aggressive benign neoplasms with a large biological spectrum. Currently, there are no reliable predictors of recurrence, malignant transformation, or metastatic behavior. Curettage is the preferred treatment option and can be performed alone or in combination with local adjuvants such as PMMA cement, alcohol, phenol, hydrogen peroxide, zinc chloride, cryoablation with liquid nitrogen, local application of zoledronic acid, and combinations. Systemic agents such as denosumab, bisphonates, or IFN-a may also be administered for effective control of the local and metastatic disease. However, even though the biology, pathophysiology, and treatment options for GCT have been extensively studied, there are still too many unanswered questions to be explored. The present article was an attempt to put essential information in one place, creating a comprehensive review that the curious reader would find interesting and enjoyable.

## Conflict of interest

No conflicts of interest are declared by any author on this article.

## References

[1] Dorfman HD, Czerniak B (1998) Bone tumors. St. Louis, Mosby.

[2] Randall RL (2003) Giant cell tumor of the sacrum. Neurosurg Focus 15(2), E13. 10.3171/foc.2003.15.2.13 15350044

[3] Campanacci M, Baldini N, Boriani S, Sudanese A (1987) Giant-cell tumor of bone. J Bone Joint Surg Am 69(1), 106–114. 3805057

[4] Ruggieri P, Mavrogenis AF, Ussia G, Angelini A, Papagelopoulos PJ, Mercuri M (2010) Recurrence after and complications associated with adjuvant treatments for sacral giant cell tumor. Clin Orthop Relat Res 468(11), 2954–2961. 20623262 10.1007/s11999-010-1448-8 PMC2947682

[5] Chawla S, Henshaw R, Seeger L, Choy E, Blay JY, Ferrari S, Kroep J, Grimer R, Reichardt P, Rutkowski P, Schuetze S, Skubitz K, Staddon A, Thomas D, Qian Y, Jacobs I (2013) Safety and efficacy of denosumab for adults and skeletally mature adolescents with giant cell tumour of bone: interim analysis of an open-label, parallel-group, phase 2 study. Lancet Oncol 14(9), 901–908. 23867211 10.1016/S1470-2045(13)70277-8

[6] Turcotte RE (2006) Giant cell tumor of bone. Orthop Clin North Am 37(1), 35–51. 16311110 10.1016/j.ocl.2005.08.005

[7] Zhang K, Chen K, Zhou M, Chen H, Lu J, Yang H (2015) Extremely large giant-cell tumor of sacrum with successful resection via posterior approach. Spine J 15(7), 1684–1685. 25666699 10.1016/j.spinee.2015.02.007

[8] Feigenberg SJ, Marcus RB Jr., Zlotecki RA, Scarborough MT, Berrey BH, Enneking WF (2003) Radiation therapy for giant cell tumors of bone. Clin Orthop Relat Res 411, 207–216. 10.1097/01.blo.0000069890.31220.b4 12782877

[9] Reid R, Banerjee S, Sciot R (2002) Giant cell tumour, in The WHO Classification of tumors. Pathology and genetics: tumors of soft tissue and bone. Fletcher D, Unni K, Mertens F, Editors Lyon, France, IARC Press.

[10] Kransdorf M, Murphey M (2009) Giant cell tumor, in The imaging of bone tumors and tumor-like lesions. Davies M, Sundaram M, James S, Editors Berlin, Heidelberg, Springer-Verlag.

[11] Dahlin DC, Cupps RE, Johnson EW Jr. (1970) Giant-cell tumor: a study of 195 cases. Cancer 25(5), 1061–1070. 4910256 10.1002/1097-0142(197005)25:5<1061::aid-cncr2820250509>3.0.co;2-e

[12] Resnick D (1995) Diagnosis of bone and joint disorders, 3rd edn Philadelphia, Saunders.

[13] Unni KK, Dahlin DC (1996) Dahlin’s bone tumors: general aspects and data on 11,087 cases, 5th edn Philadelphia, Lippincott-Raven.

[14] Resnick D, Kyriakos M, Greenway G (2002) Tumors and tumor-like lesions of bone: imaging and pathology of specific lesions, in The diagnosis of bone and joint disorders, 4th edn Resnick D, Editor Philadelphia, Saunders.

[15] Thangaraj R, Grimer RJ, Carter SR, Stirling AJ, Spilsbury J, Spooner D (2010) Giant cell tumour of the sacrum: a suggested algorithm for treatment. Eur Spine J 19(7), 1189–1194. 20076978 10.1007/s00586-009-1270-8 PMC2900020

[16] van der Heijden L, Dijkstra PD, van de Sande MA, Kroep JR, Nout RA, van Rijswijk CS, Bovée JV, Hogendoorn PC, Gelderblom H (2014) The clinical approach toward giant cell tumor of bone. Oncologist 19(5), 550–561. 24718514 10.1634/theoncologist.2013-0432 PMC4012970

[17] van der Heijden L, van de Sande MA, van der Geest IC, Schreuder HW, van Royen BJ, Jutte PC, Bramer JA, Öner FC, van Noort-Suijdendorp AP, Kroon HM, Dijkstra PD (2014) Giant cell tumors of the sacrum-a nationwide study on midterm results in 26 patients after intralesional excision. Eur Spine J 23(9), 1949–1962. 24614982 10.1007/s00586-014-3263-5

[18] Jaffe HL, Lichtenstein L, Portis RB (1940) Giant cell tumor of bone. Its pathologic appearance, grading, supposed variants and treatment. Arch Pathol 30(3), 993–1031.

[19] Enneking WF, Spanier SS, Goodman MA (1980) A system for the surgical staging of musculoskeletal sarcoma. Clin Orthop Relat Res 415, 4–18. 10.1097/01.blo.0000093891.12372.0f 14612624

[20] Abat F, Almenara M, Peiro A, Trullols L, Bague S, Gracia I (2015) Giant cell tumour of bone: a series of 97 cases with a mean follow-up of 12 years. Rev Esp Cir Ortop Traumatol 59(1), 59–65. 25151296 10.1016/j.recot.2014.06.005

[21] Wang H, Wan N, Hu Y (2012) Giant cell tumour of bone: a new evaluating system is necessary. Int Orthop 36(12), 2521–2527. 23052276 10.1007/s00264-012-1664-9 PMC3508056

[22] Levine E, De Smet AA, Neff JR (1984) Role of radiologic imaging in management planning of giant cell tumor of bone. Skeletal Radiol 12(2), 79–89. 6484605 10.1007/BF00360811

[23] Chakarun CJ, Forrester DM, Gottsegen CJ, Patel DB, White EA, Matcuk GR Jr. (2013) Giant cell tumor of bone: review, mimics, and new developments in treatment. Radiographics 33(1), 197–211. 23322837 10.1148/rg.331125089

[24] Aoki J, Tanikawa H, Ishii K, Seo GS, Karakida O, Sone S, Ichikawa T, Kachi K (1996) MR findings indicative of hemosiderin in giant-cell tumor of bone: frequency, cause, and diagnostic significance. AJR Am J Roentgenol 166(1), 145–148. 8571864 10.2214/ajr.166.1.8571864

[25] Herman SD, Mesgarzadeh M, Bonakdarpour A, Dalinka MK (1987) The role of magnetic resonance imaging in giant cell tumor of bone. Skeletal Radiol 16(8), 635–643. 3423832 10.1007/BF00357112

[26] Kaplan PA, Murphey M, Greenway G, Resnick D, Sartoris DJ, Harms S (1987) Fluid-fluid levels in giant cell tumors of bone: report of two cases. J Comput Tomogr 11(2), 151–155. 3581850 10.1016/0149-936x(87)90008-7

[27] Hudson TM, Schiebler M, Springfield DS, Enneking WF, Hawkins IF Jr., Spanier SS (1984) Radiology of giant cell tumors of bone: computed tomography, arthro-tomography, and scintigraphy. Skeletal Radiol 11(2), 85–95. 6322349 10.1007/BF00348795

[28] Anchan C (2008) Giant cell tumor of bone with secondary aneurysmal bone cyst. Int J Shoulder Surg 2(3), 68. 20300319 10.4103/0973-6042.42582 PMC2840825

[29] Levine E, De Smet AA, Neff JR, Martin NL (1984) Scintigraphic evaluation of giant cell tumor of bone. AJR Am J Roentgenol 143(2), 343–348. 6234786 10.2214/ajr.143.2.343

[30] Simon MA, Kirchner PT (1980) Scintigraphic evaluation of primary bone tumors. Comparison of technetium-99m phosphonate and gallium citrate imaging. J Bone Joint Surg Am 62(5), 758–764. 6446566

[31] Grote HJ, Braun M, Kalinski T, Pomjanski N, Back W, Bleyl U, Böcking A, Roessner A (2004) Spontaneous malignant transformation of conventional giant cell tumor. Skeletal Radiol 33(3), 169–175. 14749901 10.1007/s00256-003-0682-5

[32] Bertoni F, Bacchini P, Staals EL (2003) Malignancy in giant cell tumor of bone. Cancer 97(10), 2520–2529. 12733152 10.1002/cncr.11359

[33] Manaster BJ, Doyle AJ (1993) Giant cell tumors of bone. Radiol Clin North Am 31(2), 299–323. 8446751

[34] Nusbacher N, Sclafani SJ, Birla SR (1981) Case report 155. Polyostotic Paget disease complicated by benign giant cell tumor of left clavicle, Skeletal Radiol 6(3), 233–235. 7268470 10.1007/BF00347195

[35] Mavrogenis AF, Angelini A, Errani C, Rimondi E (2014) How should musculoskeletal biopsies be performed? Orthopedics 37(9), 585–588. 25198351 10.3928/01477447-20140825-03

[36] Rimondi E, Rossi G, Bartalena T, Ciminari R, Alberghini M, Ruggieri P, Errani C, Angelini A, Calabrò T, Abati CN, Balladelli A, Tranfaglia C, Mavrogenis AF, Vanel D, Mercuri M (2011) Percutaneous CT-guided biopsy of the musculoskeletal system: results of 2027 cases. Eur J Radiol 77(1), 34–42. 20832220 10.1016/j.ejrad.2010.06.055

[37] Thomas DM (2012) RANKL, denosumab, and giant cell tumor of bone. Curr Opin Oncol 24(4), 397–403. 22581354 10.1097/CCO.0b013e328354c129

[38] Roux S, Amazit L, Meduri G, Guiochon-Mantel A, Milgrom E, Mariette X (2002) RANK (receptor activator of nuclear factor kappa B) and RANK ligand are expressed in giant cell tumors of bone. Am J Clin Pathol 117(2), 210–216. 11863217 10.1309/BPET-F2PE-P2BD-J3P3

[39] Liao TS, Yurgelun MB, Chang SS, Zhang HZ, Murakami K, Blaine TA, Parisien MV, Kim W, Winchester RJ, Lee FY (2005) Recruitment of osteoclast precursors by stromal cell derived factor-1 (SDF-1) in giant cell tumor of bone. J Orthop Res 23(1), 203–209. 15607894 10.1016/j.orthres.2004.06.018

[40] Rao UN, Goodman M, Chung WW, Swalski P, Pal R, Finkelstein S (2005) Molecular analysis of primary and recurrent giant cell tumors of bone. Cancer Genet Cytogenet 158(2), 126–136. 15796959 10.1016/j.cancergencyto.2004.09.015

[41] Gorunova L, Vult von Steyern F, Storlazzi CT, Bjerkehagen B, Follerås G, Heim S, Mandahl N, Mertens F (2009) Cytogenetic analysis of 101 giant cell tumors of bone: nonrandom patterns of telomeric associations and other structural aberrations. Genes Chromosomes Cancer 48(7), 583–602. 19396867 10.1002/gcc.20667

[42] Forsyth RG, De Boeck G, Bekaert S, De Meyer T, Taminiau AH, Uyttendaele D, Roels H, Praet MM, Hogendoorn PC (2008) Telomere biology in giant cell tumour of bone. J Pathol 214(5), 555–563. 18278785 10.1002/path.2301

[43] Behjati S, Tarpey PS, Presneau N, Scheipl S, Pillay N, Van Loo P, Wedge DC, Cooke SL, Gundem G, Davies H, Nik-Zainal S, Martin S, McLaren S, Goody V, Robinson B, Butler A, Teague JW, Halai D, Khatri B, Myklebost O, Baumhoer D, Jundt G, Hamoudi R, Tirabosco R, Amary MF, Futreal PA, Stratton MR, Campbell PJ, Flanagan AM (2013) Distinct H3F3A and H3F3B driver mutations define chondroblastoma and giant cell tumor of bone. Nat Genet 45(12), 1479–1482. 24162739 10.1038/ng.2814 PMC3839851

[44] Oda Y, Sakamoto A, Saito T, Matsuda S, Tanaka K, Iwamoto Y, Tsuneyoshi M (2001) Secondary malignant giant-cell tumour of bone: molecular abnormalities of p53 and H-ras gene correlated with malignant transformation. Histopathology 39(6), 629–637. 11903582 10.1046/j.1365-2559.2001.01275.x

[45] Saito T, Mitomi H, Izumi H, Suehara Y, Okubo T, Torigoe T, Takagi T, Kaneko K, Sato K, Matsumoto T, Yao T (2011) A case of secondary malignant giant-cell tumor of bone with p53 mutation after long-term follow-up. Hum Pathol 42(5), 727–733. 21237496 10.1016/j.humpath.2010.08.008

[46] Balke M, Schremper L, Gebert C, Ahrens H, Streitbuerger A, Koehler G, Hardes J, Gosheger G (2008) Giant cell tumor of bone: treatment and outcome of 214 cases. J Cancer Res Clin Oncol 134(9), 969–978. 18322700 10.1007/s00432-008-0370-x PMC12160765

[47] Sanerkin NG (1980) Malignancy, aggressiveness, and recurrence in giant cell tumor of bone. Cancer 46(7), 1641–1649. 7417958 10.1002/1097-0142(19801001)46:7<1641::aid-cncr2820460725>3.0.co;2-z

[48] Murphey MD, Nomikos GC, Flemming DJ, Gannon FH, Temple HT, Kransdorf MJ (2001) From the archives of AFIP. Imaging of giant cell tumor and giant cell reparative granuloma of bone: radiologic-pathologic correlation. Radiographics 21(5), 1283–1309. 11553835 10.1148/radiographics.21.5.g01se251283

[49] Dominkus M, Ruggieri P, Bertoni F, Briccoli A, Picci P, Rocca M, Mercuri M (2006) Histologically verified lung metastases in benign giant cell tumours. 14 Cases from a single institution. Int Orthop 30(6), 499–504. 16909252 10.1007/s00264-006-0204-x PMC3172731

[50] Donthineni R, Boriani L, Ofluoglu O, Bandiera S (2009) Metastatic behaviour of giant cell tumour of the spine. Int Orthop 33(2), 497–501. 18461324 10.1007/s00264-008-0560-9 PMC2899057

[51] Siebenrock KA, Unni KK, Rock MG (1998) Giant-cell tumour of bone metastasising to the lungs. A long-term follow-up. J Bone Joint Surg Br 80(1), 43–47. 9460951 10.1302/0301-620x.80b1.7875

[52] Lewis VO. 2007 What’s new in musculoskeletal oncology. J Bone Joint Surg Am 89(6), 1399–1407. 17545444 10.2106/JBJS.G.00075

[53] Mirra JM, Picci P, Gold RH (1989) Bone tumors: clinical, radiologic, and pathologic correlations. Philadelphia, Lea & Febiger.

[54] Horvai A, Unni KK (2006) Premalignant conditions of bone. J Orthop Sci 11(4), 412–423. 16897210 10.1007/s00776-006-1037-6 PMC2780648

[55] Haskell A, Wodowoz O, Johnston JO (2003) Metachronous multicentric giant cell tumor: a case report and literature review. Clin Orthop Relat Res 412, 162–168. 10.1097/01.blo.0000068770.86536.e1 12838067

[56] Cummins CA, Scarborough MT, Enneking WF (1996) Multicentric giant cell tumor of bone. Clin Orthop Relat Res 322, 245–252. 8542701

[57] Bandyopadhyay R, Biswas S, Bandyopadhyay SK, Ray MM (2016) Synchronous multicentric giant cell tumor. J Cancer Res Ther 6(1), 106–108. 10.4103/0973-1482.63552 20479561

[58] Morey V, Sankineani SR, Kumar R (2014) Multifocal metachronous giant cell tumour in bilateral upper limb: a rare case presentation. Musculoskelet Surg 98(2), 165–169. 22990983 10.1007/s12306-012-0223-2

[59] Kafchitsas K, Habermann B, Proschek D, Kurth A, Eberhardt C (2010) Functional results after giant cell tumor operation near knee joint and the cement radiolucent zone as indicator of recurrence. Anticancer Res 30(9), 3795–3799. 20944172

[60] Zhen W, Yaotian H, Songjian L, Ge L, Qingliang W (2004) Giant-cell tumour of bone. The long-term results of treatment by curettage and bone graft. J Bone Joint Surg Br 86(2), 212–216. 15046435 10.1302/0301-620x.86b2.14362

[61] Errani C, Ruggieri P, Asenzio MA, Toscano A, Colangeli S, Rimondi E, Rossi G, Longhi A, Mercuri M (2010) Giant cell tumor of the extremity: a review of 349 cases from a single institution. Cancer Treat Rev 36(1), 1–7. 19879054 10.1016/j.ctrv.2009.09.002

[62] Salunke AA, Chen Y, Chen X, Tan JH, Singh G, Tai BC, Khin LW, Puhaindran ME (2015) Does pathological fracture affect the rate of local recurrence in patients with a giant cell tumour of bone? A meta-analysis. Bone Joint J 97-B(11), 1566–1571. 26530662 10.1302/0301-620X.97B11.35326

[63] Kivioja AH, Blomqvist C, Hietaniemi K, Trovik C, Walloe A, Bauer HC, Jorgensen PH, Bergh P, Follerås G (2008) Cement is recommended in intralesional surgery of giant cell tumors: a Scandinavian Sarcoma Group study of 294 patients followed for a median time of 5 years. Acta Orthop 79(1), 86–93. 18283578 10.1080/17453670710014815

[64] Klenke FM, Wenger DE, Inwards CY, Rose PS, Sim FH (2011) Giant cell tumor of bone: risk factors for recurrence. Clin Orthop Relat Res 469(2), 591–599. 20706812 10.1007/s11999-010-1501-7 PMC3018195

[65] Capanna R, Fabbri N, Bettelli G (1990) Curettage of giant cell tumor of bone. The effect of surgical technique and adjuvants on local recurrence rate. Chir Organi Mov 75(1 Suppl), 206. 2249534

[66] Durr HR, Maier M, Jansson V, Baur A, Refior HJ (1999) Phenol as an adjuvant for local control in the treatment of giant cell tumour of the bone. Eur J Surg Oncol 25(6), 610–618. 10556009 10.1053/ejso.1999.0716

[67] Trieb K, Bitzan P, Lang S, Dominkus M, Kotz R (2001) Recurrence of curetted and bone-grafted giant-cell tumours with and without adjuvant phenol therapy. Eur J Surg Oncol 27(2), 200–202. 11289759 10.1053/ejso.2000.1086

[68] Gaston CL, Bhumbra R, Watanuki M, Abudu AT, Carter SR, Jeys LM, Tillman RM, Grimer RJ (2011) Does the addition of cement improve the rate of local recurrence after curettage of giant cell tumours in bone? J Bone Joint Surg Br 93(12), 1665–1669. 22161931 10.1302/0301-620X.93B12.27663

[69] van der Heijden L, van de Sande MA, Dijkstra PD (2012) Soft tissue extension increases the risk of local recurrence after curettage with adjuvants for giant-cell tumor of the long bones. Acta Orthop 83(4), 401–405. 22880716 10.3109/17453674.2012.711193 PMC3427632

[70] Boons HW, Keijser LC, Schreuder HW, Pruszczynski M, Lemmens JA, Veth RP (2002) Oncologic and functional results after treatment of giant cell tumors of bone. Arch Orthop Trauma Surg 122(1), 17–23. 11995874 10.1007/s004020100317

[71] Ghert MA, Rizzo M, Harrelson JM, Scully SP (2002) Giant-cell tumor of the appendicular skeleton. Clin Orthop Relat Res 400, 201–210. 10.1097/00003086-200207000-00025 12072763

[72] Turcotte RE, Wunder JS, Isler MH, Bell RS, Schachar N, Masri BA, Moreau G, Davis AM, Canadian Sarcoma Group (2002) Giant cell tumor of long bone: a Canadian Sarcoma Group study. Clin Orthop Relat Res 397, 248–258. 10.1097/00003086-200204000-00029 11953616

[73] Ward WG, Sr, Li G III (2002) Customized treatment algorithm for giant cell tumor of bone: report of a series. Clin Orthop Relat Res 397, 259–270. 10.1097/00003086-200204000-00030 11953617

[74] O’Donnell RJ, Springfield DS, Motwani HK, Ready JE, Gebhardt MC, Mankin HJ (1994) Recurrence of giant-cell tumors of the long bones after curettage and packing with cement. J Bone Joint Surg Am 76(12), 1827–1833. 7989388 10.2106/00004623-199412000-00009

[75] Wada T, Kaya M, Nagoya S, Kawaguchi S, Isu K, Yamashita T, Yamawaki S, Ishii S (2002) Complications associated with bone cementing for the treatment of giant cell tumors of bone. J Orthop Sci 7(2), 194–198. 11956979 10.1007/s007760200033

[76] Klenke FM, Wenger DE, Inwards CY, Rose PS, Sim FH (2011) Recurrent giant cell tumor of long bones: analysis of surgical management. Clin Orthop Relat Res 469(4), 1181–1187. 20857250 10.1007/s11999-010-1560-9 PMC3048273

[77] Balke M, Ahrens H, Streitbuerger A, Koehler G, Winkelmann W, Gosheger G, Hardes J (2009) Treatment options for recurrent giant cell tumors of bone. J Cancer Res Clin Oncol 135(1), 149–158. 18521629 10.1007/s00432-008-0427-x PMC12160313

[78] Su YP, Chen WM, Chen TH (2004) Giant-cell tumors of bone: an analysis of 87 cases. Int Orthop 28(4), 239–243. 15160253 10.1007/s00264-004-0564-z PMC3456930

[79] Lin WH, Lan TY, Chen CY, Wu K, Yang RS (2011) Similar local control between phenol- and ethanol-treated giant cell tumors of bone. Clin Orthop Relat Res 469(11), 3200–3208. 21732023 10.1007/s11999-011-1962-3 PMC3183197

[80] Benevenia J, Patterson FR, Beebe KS, Abdelshahed MM, Uglialoro AD (2012) Comparison of phenol and argon beam coagulation as adjuvant therapies in the treatment of stage 2 and 3 benign-aggressive bone tumors. Orthopedics 35(3), e371–e378. 22385449 10.3928/01477447-20120222-22

[81] Malawer MM, Bickels J, Meller I, Buch RG, Henshaw RM, Kollender Y (1999) Cryosurgery in the treatment of giant cell tumor. A long-term followup study. Clin Orthop Relat Res 359, 176–188. 10.1097/00003086-199902000-00019 10078141

[82] Marcove RC, Sheth DS, Brien EW, Huvos AG, Healey JH (1994) Conservative surgery for giant cell tumors of the sacrum. The role of cryosurgery as a supplement to curettage and partial excision. Cancer 74(4), 1253–1260. 8055446 10.1002/1097-0142(19940815)74:4<1253::aid-cncr2820740412>3.0.co;2-9

[83] Schreuder HW, Keijser LC, Veth RP (1999) Beneficial effects of cryosurgical treatment in benign and low-grade-malignant bone tumors in 120 patients. Ned Tijdschr Geneeskd 143(45), 2275–2281. 10578429

[84] Marcove RC, Weis LD, Vaghaiwalla MR, Pearson R (1978) Cryosurgery in the treatment of giant cell tumors of bone: a report of 52 consecutive cases. Clin Orthop Relat Res 134, 275–289. 729255

[85] Jacobs PA, Clemency RE Jr. (1985) The closed cryosurgical treatment of giant cell tumor. Clin Orthop Relat Res 192, 149–158. 3967417

[86] Alkalay D, Kollender Y, Mozes M, Meller I (1996) Giant cell tumors with intraarticular fracture. Two-stage local excision, cryosurgery and cementation in 5 patients with distal femoral tumor followed for 2–4 years. Acta Orthop Scand 67(3), 291–294. 8686472 10.3109/17453679608994692

[87] Abdelrahman M, Bassiony AA, Shalaby H, Assal MK (2009) Cryosurgery and impaction subchondral bone graft for the treatment of giant cell tumor around the knee. HSS J 5(2), 123–128. 19590926 10.1007/s11420-009-9125-8 PMC2744763

[88] Wittig JC, Simpson BM, Bickels J, Kellar-Graney KL, Malawer MM (2001) Giant cell tumor of the hand: superior results with curettage, cryosurgery, and cementation. J Hand Surg Am 26(3), 546–555. 11418922 10.1053/jhsu.2001.22525

[89] Nishisho T, Hanaoka N, Endo K, Takahashi M, Yasui N (2011) Locally administered zoledronic acid therapy for giant cell tumor of bone. Orthopedics 34(7), e312–e315. 21717996 10.3928/01477447-20110526-22

[90] Niu X, Zhang Q, Hao L, Ding Y, Li Y, Xu H, Liu W (2012) Giant cell tumor of the extremity: retrospective analysis of 621 Chinese patients from one institution. J Bone Joint Surg Am 94(5), 461–467. 22398741 10.2106/JBJS.J.01922

[91] Moon MS, Kim SS, Moon JL, Kim SS, Moon H (2013) Treating giant cell tumours with curettage, electrocautery, burring, phenol irrigation, and cementation. J Orthop Surg (Hong Kong) 21(2), 209–212. 24014786 10.1177/230949901302100219

[92] van der Heijden L, van der Geest IC, Schreuder HW, van de Sande MA, Dijkstra PD (2014) Liquid nitrogen or phenolization for giant cell tumor of bone? A comparative cohort study of various standard treatments at two tertiary referral centers. J Bone Joint Surg Am 96(5), e35. 24599207 10.2106/JBJS.M.00516

[93] Veth R, Schreuder B, van Beem H, Pruszczynski M, de Rooy J (2005) Cryosurgery in aggressive, benign, and low-grade malignant bone tumours. Lancet Oncol 6(1), 25–34. 15629273 10.1016/S1470-2045(04)01710-3

[94] Lopez-Pousa A, Martin Broto J, Garrido T, Vazquez J (2015) Giant cell tumour of bone: new treatments in development. Clin Transl Oncol 17(6), 419–430. 25617146 10.1007/s12094-014-1268-5 PMC4448077

[95] Group ESESNW (2014) Bone sarcomas: ESMO Clinical Practice Guidelines for diagnosis, treatment and follow-up. Ann Oncol 25(Suppl 3), 113–123. 10.1093/annonc/mdu256 25210081

[96] Cheng YY, Huang L, Lee KM, Xu JK, Zheng MH, Kumta SM (2004) Bisphosphonates induce apoptosis of stromal tumor cells in giant cell tumor of bone. Calcif Tissue Int 75(1), 71–77. 15037971 10.1007/s00223-004-0120-2

[97] Tse LF, Wong KC, Kumta SM, Huang L, Chow TC, Griffith JF (2008) Bisphosphonates reduce local recurrence in extremity giant cell tumor of bone: a case-control study. Bone 42(1), 68–73. 17962092 10.1016/j.bone.2007.08.038

[98] Balke M, Campanacci L, Gebert C, Picci P, Gibbons M, Taylor R, Hogendoorn P, Kroep J, Wass J, Athanasou N (2010) Bisphosphonate treatment of aggressive primary, recurrent and metastatic giant cell tumour of bone. BMC Cancer 10, 462. 20799989 10.1186/1471-2407-10-462 PMC2940802

[99] Yasko AW (2006) Interferon therapy for giant cell tumor of bone. Curr Opinion Orthop 17(6), 568–572.

